# Application of PN Code in Time Delay Measurement of Telephone Network

**DOI:** 10.3390/s25010241

**Published:** 2025-01-03

**Authors:** Xiaozhen Jin, Yu Hua

**Affiliations:** 1 National Time Service Center, Chinese Academy of Sciences, Xi’an 710600, China; hy@ntsc.ac.cn; 2 Key Laboratory of Precise Positioning and Timing Technology, Chinese Academy of Sciences, Xi’an 710600, China

**Keywords:** telephone time service, time delay measurement, PN code, uncertainty of measurement

## Abstract

Telephone time service is a wired time service that transmits time signals through a telephone network, with the advantages of simple receiving equipment and wide coverage. But the performance of time service is not high, usually several milliseconds. The time delay measurement of the telephone network is an important factor limiting the improvement in timing performance. The existing telephone service systems measure time delay based on sending characters. The time fluctuation of modulating and demodulating characters and the low accuracy of detecting characters affect the uncertainty of time delay measurement. This article proposes a time delay measurement method based on sending PN codes. It utilizes the good autocorrelation properties of PN codes to improve detection accuracy and then improve the uncertainty of delay measurement. This article studies and designs the modulation, demodulation, and capture of PN codes. Simulation has verified that this method has strong anti-noise performance. Time delay tests have proved that this method can improve the uncertainty of delay measurement by an order of magnitude in different telephone network environments.

## 1. Introduction

Telephone time service users can obtain standard time by dialing a special phone number in the time base center. Compared with other wired time services, it has wide coverage and good safety. Compared with the Global Navigation Satellite System (GNSS) time service, it is not affected by signal obstruction and has strong anti-interference performance. Therefore, telephone time service is a better option in certain environments.

In 1988, The National Institute of Standards and Technology (NIST) in the United States began research on a telephone time service system for the first time, and launched Computer Automatic Time Service (ACTS) [1]. In 1995, the Japan Standard Time (JST) Group started a dial-up standard time service (Telephone JJY), and upgraded to “Hikari Telephone JJY” in 2024 [2]. In 1998, The National Time Service Center (NTSC) in China launched a telephone time service system [1,3], and a nationwide telephone time service system was established in 2023. In 2002, The National Physical Laboratory in India (NPLI) carried out research on telephone timing technology and made a mobile teleclock receiver and a landline teleclock receiver [4,5,6]. In 2004, The National Institute of Metrology (NIM) in Romania carried out a telephone time service experiment under DOS and WINDOWS systems [7]. To sum up, telephone time service has been widely used in many countries. The performance of time service ranges from several milliseconds to several hundred milliseconds.

The time delay of wired time service is the transmission time of the signal in the wired channel. For wired time service, such as telephone, network, and fiber, in order to make time more accurate, one-way delay (OWD) must be deducted during the time signal processing. For example, network time service uses NTP (Network Time Protocol) and PTP (Precision Time Protocol) to transmit time information, which is based on character encoding to measure time delay.

For wired time service, the key factor affecting timing performance is time delay measurement. Measurement uncertainty is a quantitative indication of the quality of time delay measurement. Uncertainty evaluation ensures that measurement results are accurate and reliable [8]. Most of the telephone time service systems in many countries are based on measuring the time delay of the telephone network by sending characters. Due to time fluctuations of modulating and demodulating characters by modems, and the low accuracy of character detection by processors, the method of sending characters reduces measurement uncertainty; it cannot accurately measure the time delay of the telephone network, making it impossible to further improve the performance of telephone time service.

PN code is a long-period digital sequence with pseudo-random noise statistical characteristics, consisting of logical 0 s and logical 1 s, which is easy to replicate. It has strong anti-noise ability and good autocorrelation properties for signal detection, and it is widely used in the field of communication, usually in GNSS time service [9,10,11,12]. In view of the shortcomings of the sending character method, this article proposes a time delay measurement method based on sending PN codes. Avoiding the process of modulating and demodulating characters, the method can transmit analog signals directly. At the same time, it uses FPGA to improve the detection accuracy of PN codes. Different measurements have proved that this method can more accurately measure telephone network time delay: it can improve the uncertainty of delay measurement by an order of magnitude. This study could form a base to improve the performance of telephone time service.

## 2. Materials and Methods

### 2.1. Measuring Time Delay Based on Sending Characters

**Figure 1:**
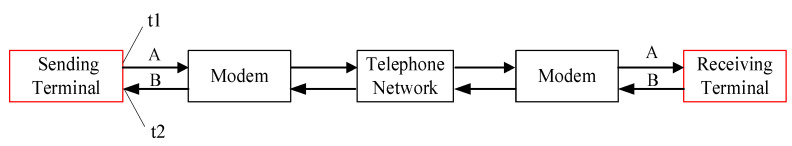
Principle of time delay measurement based on sending characters.

The principle of measuring time delay based on sending characters is shown in Figure 1 [1]. The sending terminal sends character A to the receiving terminal at time t1, and then character A is modulated by a modem and sent to the telephone network. When the receiving terminal receives character A demodulated by the modem, it immediately returns character B to the sending terminal. The sending terminal receives character B at time t2, and (t2 − t1)/2 is the time delay of the telephone network.

The delay measurement method is simple. However, the modems at both terminals may cause large time fluctuations when modulating and demodulating characters. Different modems can have a significant impact on time delay measurement [2,13]. At the same time, the processors at both terminals have a low accuracy of detecting characters, it is usually milliseconds, so the measurement uncertainty of this method is low.

### 2.2. Measuring Time Delay Based on Sending PN Codes

#### 2.2.1. Measuring Principle

**Figure 2:**
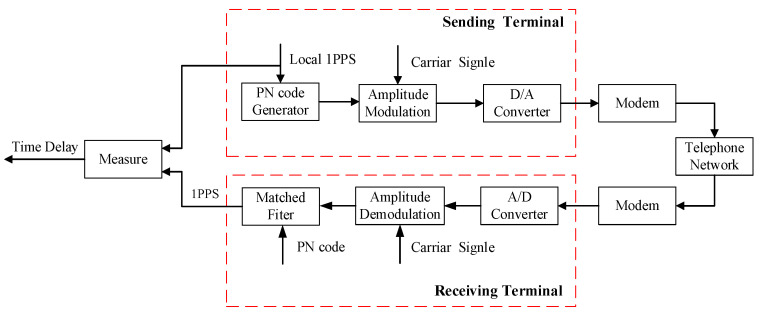
Principle of time delay measurement based on sending PN codes.

The principle of measuring time delay based on sending PN codes is shown in Figure 2. The sending terminal uses the local 1PPS signal as the trigger pulse to generate PN codes. The PN code signal is amplitude-modulated with the carrier signal. Then, the modulated signal is converted into an analog signal through a DA converter, and the analog signal is sent to a telephone network via a modem. The receiving terminal receives an analog signal through a modem, and the AD converter converts the analog signal into a digital signal. After amplitude demodulation, the digital signal is quickly captured by a matched filter [14]. A matched filter can capture PN codes and generate the maximum correlation peak in one second. The maximum correlation peak is a 1PPS signal. By measuring the rising edge of this 1PPS signal with the rising edge of the 1PPS signal from the sending terminal, we can obtain the time delay of the telephone network. The modem is no longer used for modulating and demodulating characters but for connecting an analog signal to a telephone network. Two rising edges are measured by the FPGA to improve detection accuracy.

#### 2.2.2. Design of PN Codes

The design of PN codes refers to GPS; it includes m-sequence *G*_1_ and m-sequence *G*_2,_ generated by two shift registers, and XOR operations of the two sequences can generate PN codes [15,16]. *G*_1_ and *G*_2_ can be generated by the following equation:

(1) $$\left\{\begin{array}{l} G_{1}\left(t\right)=1+t^{3}+t^{10}\\ G_{2}\left(t\right)=1+t^{2}+t^{3}+t^{6}+t^{8}+t^{9}+t^{10}\\ G\left(t\right)=G_{1}\left(t\right)⊕G_{2}\left(t+n_{i}\tau_{0}\right) \end{array}\right.$$

The length of the PN code used is 1023 bits, the repetition period of the group is 1 s, and the code rate is 1.023 kHz. $n_{i}\;$ is the phase offset between *G*_1_ and *G*_2_ and $\tau_{0}\;$ is the time corresponding to the symbol, $\tau_{0}$ = 1/1023 s.

#### 2.2.3. Modulation of PN Codes

**Figure 3:**
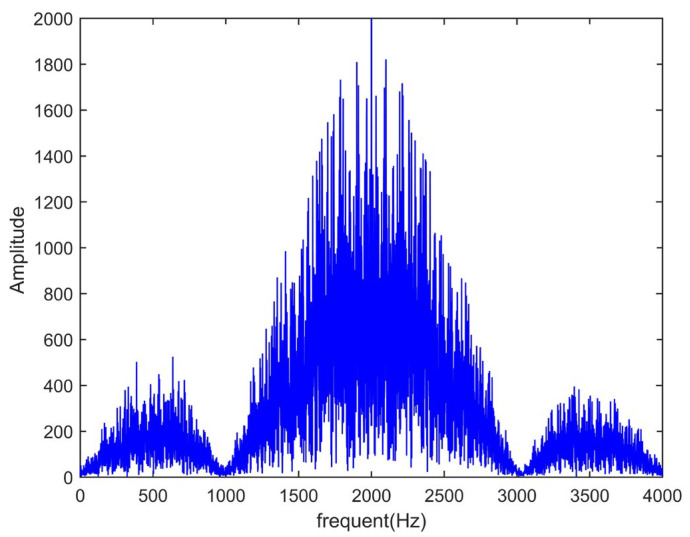
Amplitude modulation spectrum of PN codes.

The bandwidth of the telephone network is 0.3 kHz~3.4 kHz [17]. PN codes can be amplitude-modulated to meet the bandwidth. We assume that *A*(*t*) is a PN code signal and sin(*ωt*) is a sine wave signal. The frequency of *A*(*t*) is 1.023 kHz and the frequency of sin(*ωt*) is 2 kHz. The modulated signal is *A*(*t*)sin(*ωt*). According to the principle of amplitude modulation, the frequency range after modulation is 0.977 kHz~3.023 kHz. We simulate the modulated signal using MATLAB R2014b [18]. The spectrum diagram is shown in Figure 3. The horizontal axis represents the sampling frequency and the vertical axis represents the amplitude of the modulated signal.

In Figure 3, the main lobe width of the spectrum is between 0.3 kHz and 3.4 kHz. It meets the bandwidth requirement of a telephone network.

#### 2.2.4. Demodulation and Capture of PN Codes

**Figure 4:**
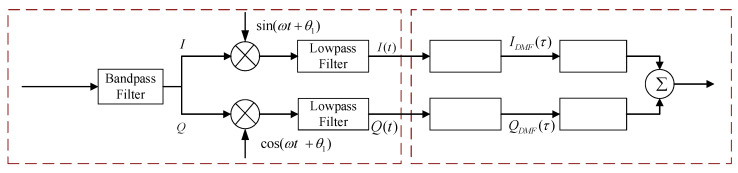
Principles of demodulating and capturing PN codes.

According to the principle of amplitude demodulation, after passing through a bandpass filter, the receiving terminal divides the modulated signal into two branches, *I* and *Q*. The *I* branch multiplies with an in-phase signal, while the *Q* branch multiplies with a quadrature signal. After passing through a low-pass filter, the signal is sent to a matched filter. The matched filter will capture the signal. Then, the signal is squared and summed, and the final output signal is a 1PPS signal. The demodulation and capture principles of PN codes are shown in Figure 4 [19,20].

1. Demodulation of PN codes:

According to Section 2.2.3, the signal from the sending terminal is *A*(*t*)sin(*ωt*). When the signal passes through the telephone network, there will be amplitude changes caused by various repeaters, phase changes caused by transmission time, and various additive noises, so we assume that the signal output by the bandpass filter is KA(t+Δt)sin(ωt+θ)+n(t). In the formula, *K* is the amplitude value after passing through the telephone network, Δ*t* is the time delay of *A*(*t*) after passing through the network, θ  is the phase difference of sin(*ωt*) after passing through the network, and n(t) is the noise in the telephone network. Assuming the phase difference between the startup time of the receiving and transmitting ends is $\;\theta^{\prime }$, the *I* branch and *Q* branch complete demodulation by multiplying with the in-phase signal $\sin\left(\omega t+\theta \prime \right)$ and quadrature signal $\cos\left(\omega t+\theta \prime \right)$. The equation is as follows:

(2) $$KA\left(t+∆t\right)\sin\left(\omega t+\theta \right)\sin\left(\omega t+\theta \prime \right)=0.5KA\left(t+∆t\right)[\cos\left(\theta -\theta^{\prime }\right)-\cos\left(2\omega t+\theta +\theta^{\prime }\right)]$$

(3) $$KA\left(t+∆t\right)\sin\left(\omega t+\theta \right)\cos\left(\omega t+\theta \prime \right)=0.5KA\left(t+∆t\right)[\sin\left(\theta -\theta^{\prime }\right)+\sin\left(2\omega t+\theta +\theta^{\prime }\right)]$$

After low-pass filtering, we can obtain I(t) and Q(t). Since noise signal still exists at this time, it is defined as n′(t).

(4) $$I\left(t\right)=0.5KA\left(t+∆t\right)\cos\left(\theta -\theta^{\prime }\right)+n\prime (t)$$

(5) $$Q\left(t\right)=0.5KA\left(t+∆t\right)\sin\left(\theta -\theta^{\prime }\right)+n\prime (t)$$

2. Capture of PN code:

The matched filter is the best linear filter with the highest signal-to-noise ratio; it plays an important role in electronic information systems such as sonar and radar [21,22,23]. Compared with the sliding correlation method, the matched filter method has a complex structure but a shorter capture time, and it is suitable for the time delay measurement of telephone time service. The following text will analyze the principle of capturing PN codes based on the matched filter method.

The output equation of the matched filter is as follows [24]:

(6) $$S_{O}=s\left(t\right)*h\left(t\right)=\int_{-\infty }^{\infty }s\left(t-\tau \right)h\left(\tau \right)d\tau =KR(t-t_{0})$$

where *s*(*t*) is the input signal, *h*(*t*) is the impulse response signal, and τ  is the phase shift of the impulse response function. After operating autocorrelation on I(t) and Q(t), we can obtain the following:

(7) $$\begin{array}{l} {\;I}_{DMF}\left(\tau \right)=\frac{1}{NT_{L}}\int_{0}^{NT_{L}}I\left(t\right)A\left(t+\tau \right)dt\\ =\frac{0.5Kcos\left(\theta -\theta^{\prime }\right)}{NT_{L}}\int_{0}^{NT_{L}}A\left(t+∆t\right)A\left(t+\tau \right)dt+\frac{0.5Kcos\left(\theta -\theta^{\prime }\right)}{NT_{L}}\int_{0}^{NT_{L}}n\prime \left(t\right)A\left(t+\tau \right)dt \end{array}$$

(8) $$\begin{array}{l} Q_{DMF}\left(\tau \right)=\frac{1}{NT_{L}}\int_{0}^{NT_{L}}Q\left(t\right)A\left(t+\tau \right)dt\\ =\frac{0.5Ksin\left(\theta -\theta^{\prime }\right)}{NT_{L}}\int_{0}^{NT_{L}}A\left(t+∆t\right)A\left(t+\tau \right)dt+\frac{0.5Ksin\left(\theta -\theta^{\prime }\right)}{NT_{L}}\int_{0}^{NT_{L}}n\prime \left(t\right)A\left(t+\tau \right)dt \end{array}$$

*N* is the length of PN codes and $T_{L}\;$ is code period, PN codes are not correlated with noises, $0.5Kcos\left(\theta -\theta^{\prime }\right)\int_{0}^{NT_{L}}n\prime \left(t\right)A\left(t+\tau \right)dt/NT_{L}\;$ and $0.5Ksin\left(\theta -\theta^{\prime }\right)\int_{0}^{NT_{L}}n\prime \left(t\right)A\left(t+\tau \right)dt/NT_{L}$ are very small. $I\left(t\right)$ and $Q\left(t\right)$ are close to the autocorrelation operation of PN codes.

The expression of PN code-related functions is as follows [24]:

(9) $$R\left(\tau \right)=\left\{\begin{array}{c} 1-\frac{m+1}{T}\left|\tau -iT\right|,0\leq \left|\tau -iT\right|\leq \frac{T}{m},i=0,1,2\ldots \\ -\frac{1}{m}\;\;\;\;\;\;\;\;\;\;\;\;\;\;\;others \end{array}\right.$$

From Equation (9), m is the period of the PN code. When τ=iT, R(τ) takes the maximum value. The code period *T* is 1 s, so a maximum correlation peak is generated every second. Combining Equations (7)–(9), the expression for the output function of the *I* and *Q* branches after autocorrelation can be written as follows:

(10) $$\begin{array}{l} I_{DMF}\left(\tau \right)=\\ \left\{\begin{array}{l} 0.5Kcos(\theta -\theta^{\prime })*(1-\frac{N+1}{NT_{L}}\left|\tau -iT-∆t\right|),0\leq \left|\tau -iT-∆t\right|\leq T_{L},i=0,1,2\ldots \\ -\frac{0.5Kcos(\theta -\theta^{\prime })}{N}\;\;\;\;\;\;\;\;\;\;\;\;\;\;\;\;\;\;\;\;\;\;others \end{array}\right. \end{array}$$

(11) $$\begin{array}{l} Q_{DMF}\left(\tau \right)=\\ \left\{\begin{array}{l} 0.5Ksin(\theta -\theta^{\prime })*(1-\frac{N+1}{NT_{L}}\left|\tau -iT-∆t\right|),0\leq \left|\tau -iT-∆t\right|\leq T_{L},i=0,1,2\ldots \\ -\frac{0.5Ksin(\theta -\theta^{\prime })}{N}\;\;\;\;\;\;\;\;\;\;\;\;\;\;\;\;\;\;\;\;\;\;others \end{array}\right. \end{array}$$

**Figure 5:**
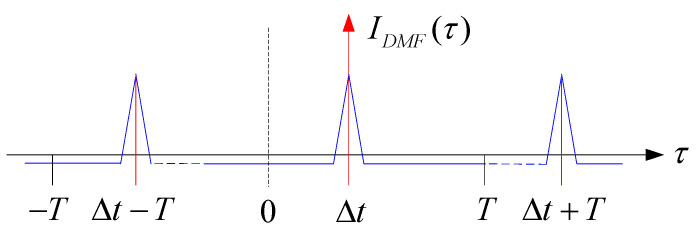
Output function curve of branch *I*.

Taking branch *I* as an example, the output function curve can be drawn according to Equation (10), as shown in Figure 5.

Assuming the 0 o’clock position in Figure 5 is the starting time for the sending terminal to send the PN code, the PN code period is *T*. After transmission through the telephone network, the output function *I_DMF_*(*τ*) of the matched filter is the maximum correlation value when τ=Δt+iT. The maximum correlation value is $0.5Kcos\left(\theta -\theta^{\prime }\right)$ and the value at other times is $0.5Kcos\left(\theta -\theta^{\prime }\right)/N$. In this article, N = 1023, and the output function *IDMF*(*τ*) tends to the impulse function, so the transmission time delay Δt can be calculated by comparing the 1PPS signal at the 0 o’clock position with the 1PPS signal generated at the time of the maximum correlation value, the period *T = NT_L_*, which is 1 s. Therefore, the time delay value can be calculated every second by capturing the relevant peak values. It can be seen from Equations (10) and (11) that the maximum correlation value is not fixed; it is affected by the phase difference (θ−θ′). Due to different startup times of the receiving terminal, the maximum correlation values output by *I_DMF_*(*τ*) and *Q_DMF_*(*τ*) are different. Even if one of the *I* and *Q* branches is zero, the maximum correlation value cannot be obtained. By summing the squares of *I_DMF_*(*τ*) and *Q_DMF_*(*τ*), this effect can be eliminated. The expression can be written as follows:

(12) $${I_{DMF}\left(\tau \right)}^{2}+{Q_{DMF}\left(\tau \right)}^{2}=\left\{\begin{array}{c} 0.25K^{2},\tau =∆t+iT,i=0,1,2\ldots \\ \frac{0.25K^{2}}{N^{2}}\;\;\;\;\;\;\;\;\;\;\;\;\;\;\;others \end{array}\right.$$

Equation (12) shows that the maximum correlation value of the sum of squares is a constant value, and the peak amplitude is only affected by the amplitude of the received signal; it will not change with the startup time.

### 2.3. Materials

The main components used in the article are FPGA and MODEM, FPGA (Intel Corporation, Santa Clara, CA, USA), MODEM (Embercom Technology Co., Ltd., Shenzhen, China).

## 3. Results

### 3.1. Anti-Noise Performance

**Figure 6:**
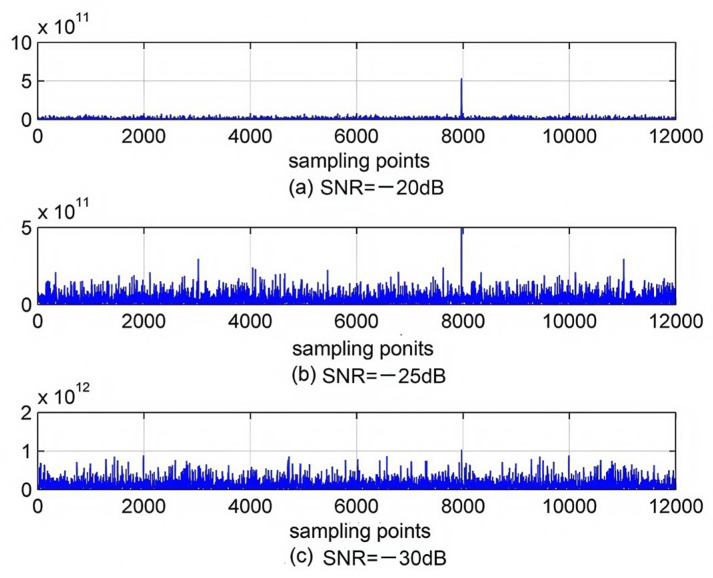
Capture results under different signal-to-noise ratios.

According to the principle described in Section 2.2, we simulated the modulation, demodulation, and capture of PN codes using MATLAB, and used Gaussian white noise instead of noise in the telephone network to verify the anti-noise performance of measuring time delay based on sending PN codes; we added white noise signals with signal-to-noise ratios of −20 dB, −25 dB, and −30 dB, and analyzed the anti-noise performance through relevant peaks. The sampling rate was set to 8 kHz and the sampling time was set to 1.5 s. The capture result of PN codes is shown in Figure 6. The horizontal axis represents the number of sampling points, and the vertical axis represents the amplitude.

From the top and middle figures, it can be seen that clear correlation peaks can be obtained at signal-to-noise ratios of −20 dB and −25 dB. This indicates that the receiving terminal accurately captures the PN codes. When the signal-to-noise ratio is below −30 dB, it is impossible to capture the PN codes. The signal-to-noise ratio in the telephone network is about 30 dB. However, when the signal-to-noise ratio is −25 dB, relevant peaks can be detected, which indicates that measuring time delay based on sending PN codes has strong anti-noise performance.

### 3.2. Experimental Verification

#### 3.2.1. Verification Method

**Figure 7:**
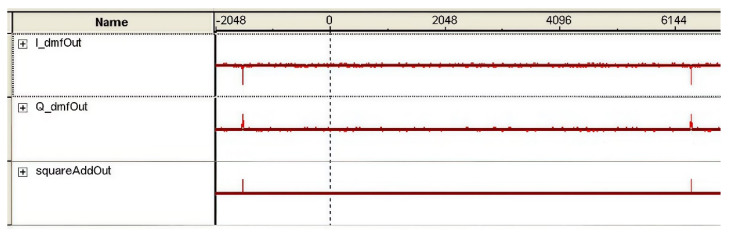
Capture of PN codes.

1. Capture verification:

The programming software for FPGA is Quartus II 9.0, We verify the capture of PN codes using the online logic analyzer included in Quartus II, and validate the effectiveness of the method. The display result of the online logic analyzer is shown in Figure 7. We can clearly see the relevant peaks.

The sampling rate of the online logic analyzer in Figure is set to 8 kHz, I_dmfOut is the output of the *I* branch matched filter, Q_dmfOut is the output of the *Q* branch matched filter, and squareAddOut is the sum of the squares of the *I* and *Q* branches. The peak interval in the figure is 8000 points. The receiving terminal can capture the PN codes sent by the sending terminal every second, generating a 1PPS signal. The receiving terminal can demodulate and capture the delay measurement signal. Furthermore, it has been verified that measuring time delay based on sending PN codes is feasible.

**Figure 8:**
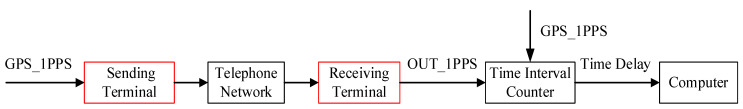
Test platform.

2. Test platform:

The test platform is shown in Figure 8. The modem is included in both the sending terminal and receiving terminal. The test can measure the one-way delay of the telephone network. The GPS_1PPS signal output by the GPS receiver serves as the reference for both terminals. The GPS_1PPS signal of GPS is used as a trigger signal for sending characters or PN codes. The receiving terminal will generate an OUT_1PPS signal after receiving characters or PN codes. The OUT_1PPS signal generated by sending PN codes is the maximum correlation peak. The OUT_1PPS signal is compared with the GPS_1PPS signal through a time interval counter, which is equivalent to comparing with the starting signal of the sending terminal. The difference between OUT_1PPS and GPS_1PPS is the time delay value of the telephone network. The value can be collected by a computer. The synchronization error of GPS_1PPS signals from both terminals is nanoseconds, so it can be ignored.

#### 3.2.2. Comparison of Two Methods

**Figure 9:**
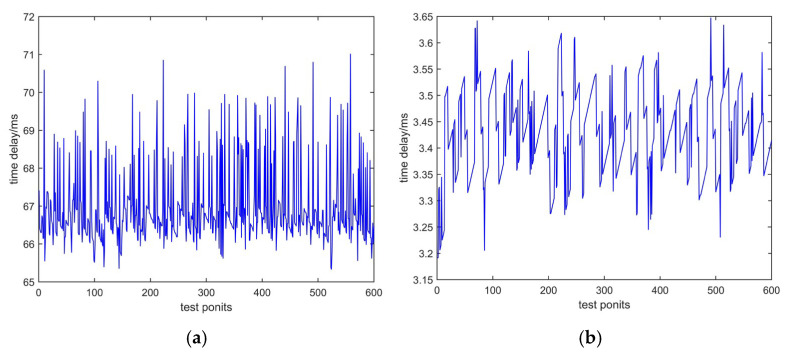
Test data of the same telephone exchange: (**a**) time delay based on sending characters; (**b**) time delay based on sending PN codes.

According to the principle shown in Figure 8, we conducted tests in the laboratory to measure time delay based on sending characters and sending PN codes. Two tests used the same phone number and passed through the same telephone exchange. The testing time was 10 min, with 600 test points. The test data are shown in Figure 9a and Figure 9b, respectively. The horizontal axis represents the number of tests, and the vertical axis represents time delay, measured in milliseconds.

Measurement uncertainty is an important indicator for evaluating the accuracy of measurements [25]. It is usually divided into Type A and Type B: Type B is related to measurement instruments and the measurement method. In this test, the measurement instruments and measurement method are the same, so we do not calculate Type B; we only calculate Type A.

Type A evaluation of standard uncertainty includes mean and standard deviation, The mean is expressed as Equation (13), which represents the arithmetic mean $\bar{q}\;$ of *n* independent observation results.

(13) $$\bar{q}=\frac{1}{n}\sum_{k=1}^{n}q_{k}$$

The standard deviation is expressed as Equation (14).

(14) $$s^{2}(q_{k})=\frac{1}{n-1}\sum_{j=1}^{n}{\left(q_{j}-\bar{q}\right)}^{2}$$

When using the arithmetic mean $\bar{q}$ as the estimate value of the measurand, the measurement uncertainty is expressed as Equation (15) [25,26,27,28].

(15) $$s(\bar{q})=\frac{s(q_{k})}{\sqrt{n}}$$

According to Equations (13) and (15), we can calculate that the mean values and the uncertainty values; they are shown in Table 1.

From Table 1, we can see that time delay based on sending PN codes can increase measurement uncertainty by one order of magnitude. This indicates that the method can more accurately measure the time delay of a telephone network.

In Figure 9, the mean values of the two methods differ significantly. The reason is that there is a significant time delay caused by the modulation and demodulation of characters via a modem when sending characters. There is no modulation or demodulation process involved in sending PN codes, so the mean of time delay is small.

#### 3.2.3. Further Verification

According to the experimental principle shown in Figure 8, we conducted separate tests based on different telephone networks in the same city and different cities. The test time was also 10 min.

**Figure 10:**
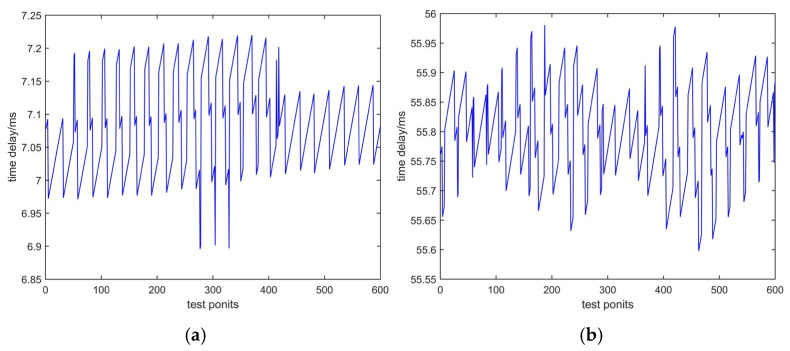
Test data of the same city: (**a**) time delay based on PSTN; (**b**) time delay based on a hybrid network of PSTN and VOIP.

1. Test based on different telephone networks in the same city:

We conducted time delay tests in Xi’an based on the PSTN network and based on the hybrid network of PSTN and VOIP. Figure 10a shows the test data based on PSTN from Lintong District to Beilin District in Xi’an and Figure 10b shows the test data based on a hybrid network of PSTN and VIOP from Lintong District to Yanta District in Xi’an.

**Figure 11:**
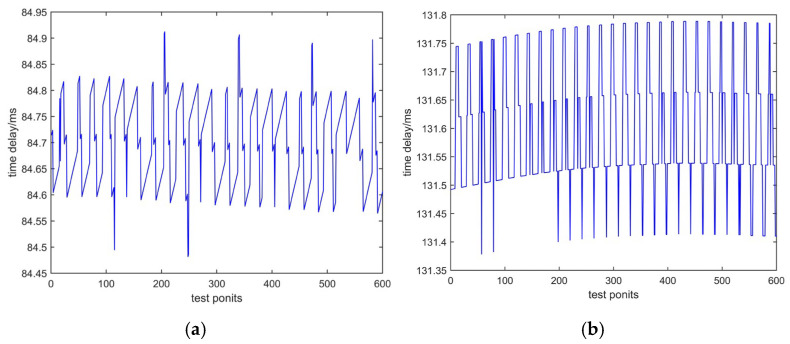
Test data of different cities: (**a**) time delay from Xi’an to Weinan; (**b**) time delay from Xi’an to Tianjin.

2. Measurement in different cities:

We measured the time delay of a telephone network from Xi’an to Weinan; they are different cities in the same province, and the distance between two cities is about 50 km. The test data are shown in Figure 11a. And we measured the time delay from Xi’an to Tianjin in different provinces. The distance between two cities is about 1000 km, and the test data are shown in Figure 11b.

Based on the four figures above and Equations (13) and (15), the measurement mean and uncertainty can be represented in Table 2.

From Table 2, we can see that the mean value of time delay varies with different networks and regions. Based on the PSTN network, the mean will increase with distance. Compared to sending characters, the uncertainty can be increased by an order of magnitude within 10 μs. This proves the feasibility of the method. The increase in uncertainty values indicates that sending PN codes can more accurately measure the latency of telephone networks.

## 4. Discussion

The purpose of this article is to measure the time delay of telephone networks more accurately, and to lay the foundation for improving telephone time service performance. Many countries measure telephone network time delay based on sending characters. This method has two disadvantages: first, using an ARM or microcontroller to detect characters results in low detection accuracy; second, the modulation and demodulation of characters by a modem may cause time delay fluctuations, and different modems may cause inconsistent fluctuations.

In response to these two disadvantages, this article measures time delay based on sending PN codes. First, using FPGA instead of ARM or a microcontroller improve detection accuracy; second, it directly transmits analog signals without using modems to modulate and demodulate characters, so it avoids time delay fluctuations. Based on the measurement uncertainty of the two methods, this method can measure the time delay of telephone networks more accurately. Some articles use standard deviation as measurement uncertainty; using standard deviation calculation can also increase uncertainty by an order of magnitude through this method.

However, compared to the sending character method, measuring time delay by sending PN codes is more complex and requires a more advanced processor. Furthermore, telephone time service requires not only measuring time delay but also transmitting time information, which also needs to be designed based on an analog signal.

Measuring time delay is a key step in telephone time service, and this study can more accurately measure time delay. Currently, the telephone timing performance in various countries ranges from several milliseconds to several hundred milliseconds. Based on the research method in this paper, it is possible to achieve the performance of telephone time service within one millisecond. The next step is measuring time delay based on sending PN codes within the national telephone network and comparing the time delay with the method of sending characters. The test can verify the universality of the method. We will also study other time delay measurement methods.

## 5. Conclusions

At present, the application of GNSS time service is very common, but in the case of signal obstruction or interference, telephone time service is a better choice. This article proposes a method for measuring time delay by sending PN codes. PN codes can be modulated with a 2 kHz sine wave to suit transmission in telephone networks. A matching filter performs a correlation operation on the received delay measurement signal, and it can efficiently capture PN codes and generate a correlation peak per second. Through simulation verification, relevant peaks can still be captured in the case of −25 dB. The method has strong anti-noise performance.

Verification experiments were conducted in the laboratory and field, and the test results showed that the fluctuation caused by the delay of sending PN codes was much smaller than the delay caused by sending characters. The PSTN network and the PSTN and VOIP hybrid network in the same city have achieved good results, as well as in different provinces and cities. In different network environments, uncertainty can be increased by an order of magnitude. The improvement in uncertainty in time delay measurement helps to provide more accurate telephone timing services. The next step is to use an analog signal to transmit time information and develop high-precision telephone time service equipment. Telephone time service will become a good backup for GNSS time service.

## Figures and Tables

**Table 1:** Time delay value of laboratory test.

Method	Mean (ms)	Uncertainty (μs)
Based on sending characters	66.86	42.68
Based on sending PN codes	3.43	3.31

**Table 2:** Time delay value of field test.

Test Place	Telephone Network	Mean (ms)	Uncertainty (μs)
Same city	PSTN	7.08	2.88
	PSTN and VIOP	55.80	3.16
Different cities	PSTN (Xi’an–Weinan)	84.69	3.35
	PSTN (Xi’an–Tianjin)	131.60	4.46

## Data Availability

The original contributions presented in this study are included in the article; further inquiries can be directed to the corresponding author.
